# *In vitro* assessment of foot-and-mouth disease vaccine immunogenicity: advances, challenges, and ethical considerations

**DOI:** 10.3389/fvets.2026.1791118

**Published:** 2026-05-01

**Authors:** Lebogang Othusitse, Tshephang I. Kabelo, Kaone A. Nkwe, LaToya Seoke, Elliot M. Fana, Kebaneilwe Lebani

**Affiliations:** 1 Botswana Vaccine Institute, Gaborone, Botswana; 2 Department of Biological Sciences, University of Reading, Reading, United Kingdom; 3 Department of Biological Sciences and Biotechnology, Faculty of Science, Botswana International University of Science and Technology, Palapye, Botswana

**Keywords:** foot-and-mouth disease, *in vitro* testing, *in vivo* testing, vaccine development, vaccine effect evaluation, immunogenicity

## Abstract

Vaccination is widely used to control foot-and-mouth disease (FMD), necessitating robust approaches for evaluating vaccine potency to ensure a consistent supply of effective and safe vaccines. Traditional (*in vivo*) potency testing methods rely on live animal models, raising ethical concerns, logistical constraints, procedural variability and significant financial costs. *In vitro* approaches have therefore been increasingly explored to complement these methods and address these challenges. Recent advancements in *in vitro* methodologies have focused on assessing vaccine-induced immune responses and virus-neutralizing activity using a variety of innovative cell-based assays, recombinant protein-based immunoassays, and molecular techniques, all of which have shown promise as surrogate tools that support vaccine potency evaluation. However, several challenges persist in optimizing these *in vitro* methods. Key obstacles include the standardization of assay protocols, ensuring reproducibility across different laboratories, and addressing the complexities of immune responses that occur *in vivo,* which are difficult to replicate in *in vitro* environments. Furthermore, there remains a need for robust validation of *in vitro* assays using a broad range of field isolates to ensure their relevance to diverse viral strains. This review examines the current landscape of *in vitro* approaches supporting FMD vaccine-induced immune responses, detailing both the advancements made in assay development and the challenges that remain to be overcome. Furthermore, there is discussion of the role of these methods in complementing conventional animal-based testing, particularly as non-invasive endpoints aligned with the principles of the 3Rs (replacement, reduction, and refinement), with potential to improve efficiency, ethical standards, and global FMD control strategies.

## 1 Introduction

Foot-and-mouth disease (FMD) is a highly contagious and economically consequential viral disease that affects a wide range of artiodactyls, including domesticated species such as cattle, sheep, goats, pigs, and water buffalo, which may occur in both farmed and wildlife populations (1). It also affects various wildlife species, including deer, antelope, African buffalo and bison (2). The economic repercussions of FMD are substantial, with global losses in endemic regions estimated at US$6.5–21 billion annually (3). These losses result from reduced livestock productivity, high morbidity, considerable mortality among young animals, disruptions to international trade, and the considerable costs of disease surveillance and control measures. More broadly, FMD outbreaks exert cascading effects on food security, export market access, and economic resilience, particularly in regions where livestock production constitutes a vital component of rural livelihoods and nutritional security (4). For instance, in Botswana, where up to 80% of agricultural income is derived from beef production and approximately 70% of beef exports are destined for the European Union (EU) market, the economic impact of FMD is particularly severe (5). Trade restrictions imposed during outbreaks can therefore significantly reduce national revenue and destabilize the livestock sector.

**Figure 1:**
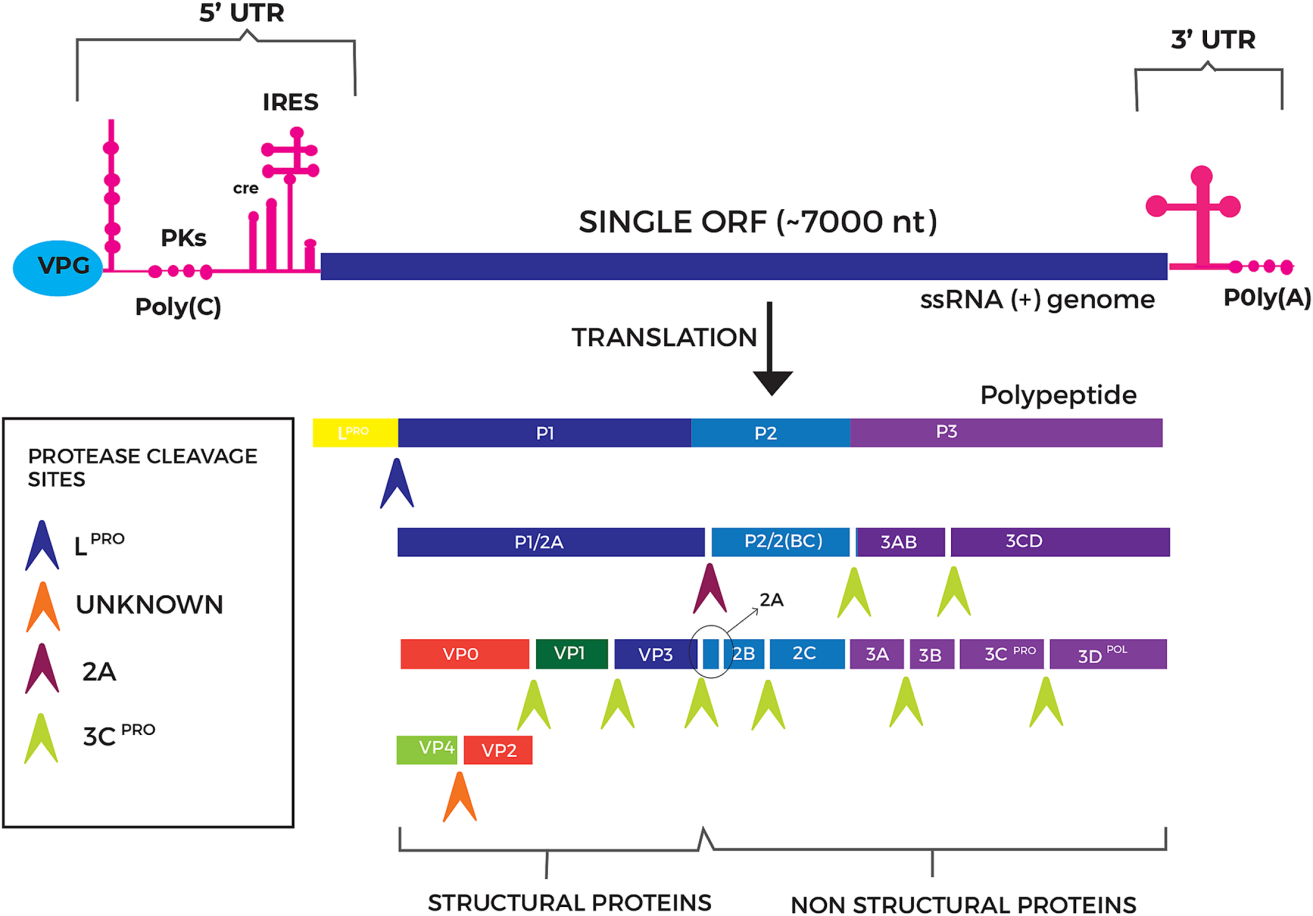
Schematic representation of the genomic organization of the foot-and-mouth disease virus (FMDV). The positive-sense single-stranded RNA genome (~8.4 kb) encodes a single large open reading frame (ORF) of ~7,000 nucleotides flanked by structured untranslated regions (UTRs). The 5′ UTR contains an internal ribosome entry site (IRES) that enables cap-independent translation initiation, while the 3′ UTR contributes to genome replication and stability (90). The ORF encodes a single polyprotein, cleaved into four structural proteins (VP1–VP4) that assemble into a pseudo-T = 3 icosahedral capsid (~25–30 nm in diameter) and act as key antigenic targets for immune recognition. The polyprotein is also processed into 8–10 non-structural proteins (L^pro^, 2A, 2B, 2C, 3A, 3B1-3, 3C^pro^, and 3D^pol^) that mediate viral replication and immune evasion.

Clinically, FMD is typically characterized by pyrexia, followed by the development of vesicular lesions and subsequent ulcerations in the oral cavity, on the feet (particularly the coronary bands and interdigital spaces), and on the mammary glands (6). These lesions result in lameness, salivation, reduced feed intake, decreased milk production, and weight loss (7). The severity and presentation of FMD clinical signs, however, vary considerably depending on several factors, including the host species, the age and immune status of the animal, as well as the specific viral strain involved (8). The etiological agent of FMD, FMD virus (FMDV), is a positive-sense, single-stranded ribonucleic acid (RNA) virus belonging to the genus *Aphthovirus* within the *Picornaviridae* virus family (9, 10). The FMDV genome, approximately 8.4 kilobases in length (Figure 1), encodes a single polyprotein that undergoes post-translational cleavage to yield four structural proteins (VP1, VP2, VP3, VP4) and 8–10 non-structural proteins (L^pro^, 2A, 2B, 2C, 3A, 3B1-3, 3C^pro^, and 3D^pol^) and some intermediates (11). The structural proteins are responsible for capsid formation and are the primary targets for confirmatory diagnostic tests such as enzyme-linked immunosorbent assays (ELISAs) and virus neutralization tests (VNTs), used in the detection and identification of antibodies against FMDV elicited by infections and vaccination (12). On the other hand, non-structural proteins are essential for viral replication, host immune evasion and are critical in the context of diagnostic tests for active and previous FMD infections (13). The detection of antibodies to non-structural proteins in an animal enables differentiation between animals that are naturally infected with live virus and those that have been vaccinated with inactivated and purified FMDV (14).

FMDV is classified into seven immunologically distinct serotypes; O, A, C, Asia-1, and the Southern African Territories (SAT) serotypes: SAT 1, SAT 2, and SAT 3. Each of which encompasses a diverse array of antigenically unique topotypes and lineages (15). Despite sharing conserved genomic regions, FMDV serotypes exhibit substantial antigenic differences, particularly in the capsid proteins, that prevent cross-protection and justify their classification into seven immunologically distinct groups. These serotypes differ in their geographic distribution and pose distinct epidemiological implications (16). Within each serotype, additional genetic and antigenic diversity gives rise to topotypes which are regionally confined viral lineages defined by greater than 15% nucleotide divergence in the VP1 coding region. The presence of multiple topotypes within a single serotype presents a significant challenge for vaccine development and implementation, as protective immunity tends to be topotype-specific (17).

Vaccination remains the cornerstone of FMD control in endemic regions, with vaccine efficacy largely dependent on antigenic compatibility with circulating field strains (16). This compatibility is critical, as protective immunity relies on the presentation of immunodominant epitopes particularly those on viral capsid proteins such as VP1 which are the primary target of neutralizing antibodies (18). Accordingly, the selection of vaccine strains requires careful antigenic characterization to ensure that key structural epitopes are preserved, thereby eliciting a robust and FMDV specific immune response. When these epitopes are accurately matched to those of the circulating FMVD strain, the vaccine can induce the production of virus-specific neutralizing antibodies that bind to the FMDV and inhibit its replication, effectively preventing infection in both domestic and wildlife populations (19). In contrast, antigenic mismatch between vaccine strains and circulating field viruses may result in suboptimal immune responses, thereby compromising protective immunity and facilitating continued transmission. This can occur when genetic mutations in the circulating virus alter key structural epitopes, reducing the effectiveness of the existing vaccine formulation (20). Therefore, continuous surveillance of circulating virus strains and timely adjustments to vaccine formulations are vital for maintaining effective control of FMD and curbing its transmission in affected areas (16).

It is important to distinguish between product potency and immunogenicity in the context of vaccine evaluation. Product potency, as defined in regulatory frameworks, refers to a quantitative measure of vaccine quality, typically expressed in terms of antigen content, dose, or biological activity (21). High-potency vaccines have been shown to confer protection even against heterologous FMDV strains, demonstrating the functional relevance of potency in eliciting protective immunity (22). In contrast, immunogenicity reflects the ability of a vaccine to elicit an immune response, often measured through antibody titers, neutralization capacity, or molecular markers (23). While immunogenicity is closely associated with vaccine performance, it does not constitute a direct measure of product potency. Vaccine potency, safety, and efficacy are considered critical quality attributes (CQAs) that define the overall quality, regulatory acceptability, and functional performance of FMD vaccines (21).

The potency of FMD vaccines is traditionally assessed through *in vivo* testing (24), typically involving challenge and protection studies designed to determine protective dose or the proportion of animals protected following exposure. However, these conventional methods present several limitations. As emphasized by Kiani et al. (25), the methods are resource-intensive and time-consuming, requiring substantial investment in animal housing, monitoring and data collection. In addition, ethical concerns associated with the use of live animals, particularly the induction of disease endpoints, have become increasingly prominent in the context of evolving animal welfare standards.

In this context, *in vitro* approaches for vaccine evaluation have gained increasing attention for their role in supporting and refining traditional *in vivo* methods. These techniques, which utilize cultured cells, molecular platforms, and immunological assays, offer advantages in terms of cost, scalability, and turnaround time (26). Importantly, they support the principles of the 3Rs (replacement, reduction, and refinement), which aim to promote more humane and sustainable research practices (27). While some *in vitro* systems represent a pathway toward replacement, many currently applied approaches particularly immunological assays such as ELISA still rely on animal-derived materials (e.g., sera) and are therefore more accurately aligned with the principle of refinement. These methods reflect a shift from invasive clinical endpoints, such as disease severity and mortality, to less invasive immunological and molecular readouts, including antibody titers and viral load measurements. Furthermore, such approaches contribute to reduction by enabling more efficient experimental designs and minimizing the number of animals required for vaccine evaluation (28).

Many of the *in vitro* methods discussed in this review primarily assess vaccine-induced immune responses and may therefore serve as surrogate or supportive indicators within vaccine potency evaluation frameworks, rather than as standalone measures of product potency. Their integration into vaccine testing strategies is particularly valuable in improving data reproducibility, enhancing standardization, and enabling high-throughput screening of vaccine candidates. This review aims to examine the current challenges associated with *in vitro* approaches for assessing FMD vaccine-induced immune responses, the advancements made in developing reliable immunological and molecular assays, and the potential directions for future research in this domain. Given the critical need for effective control strategies, the continued development and validation of innovative, non-live animal and minimally invasive testing approaches will play a pivotal role in enhancing vaccine evaluation processes and supporting the long-term goal of reducing the economic and epidemiological impact of FMD.

## 2 *In vivo* methods for FMD vaccine potency assessment: standards, assays and limitations

FMD vaccine potency in artiodactyls is primarily evaluated based on internationally recognized standards outlined by the World Organisation for Animal Health (WOAH), particularly those described in the WOAH Terrestrial Manual (21). In addition to WOAH standards, many countries implement supplementary national or regional guidelines that may incorporate additional testing requirements, locally circulating virus strains, or regulatory criteria adapted to specific epidemiological contexts and vaccine production practices. The WOAH standards utilize two key *in vivo* tests, the Protective Group Proportion (PGP) test and the 50% protective dose (PD₅₀) test which serve to assess both the clinical efficacy and immune response elicited by the vaccine (21).

The PGP test, as described by the WOAH (13), evaluates the clinical efficacy of an FMD vaccine by exposing both vaccinated and unvaccinated control animals to virulent FMDV under controlled conditions. Clinical protection is evaluated by comparing the proportion of vaccinated animals that remain clinically unaffected or exhibit only mild signs (e.g., vesicular lesions, pyrexia, or lameness), relative to the clinical outcomes observed in unvaccinated controls. According to the European Pharmacopoeia, a vaccine is considered potent if it produces a statistically significant reduction in the severity and incidence of these clinical signs relative to unvaccinated controls (29). This test thus ensures that the vaccine not only induces an immune response but also mitigates the clinical manifestations of disease in a real-world virus exposure scenario.

In contrast to the PGP test, the PD₅₀ test provides a quantitative, biologically-based measure of vaccine potency by determining the dose required to protect 50% of vaccinated animals from clinical disease (21). This test involves vaccinating groups of animals with graded doses of the vaccine, followed by a challenge with virulent FMDV and then observing the proportion protected in each group. A minimum potency of 3 PD₅₀ per dose is generally required for routine vaccination, while emergency or high-potency vaccines must meet a homologous potency of 6 PD₅₀, as recommended by the WOAH (13). In addition to evaluating protection outcomes, the PD₅₀ test also reflects the neutralizing antibody response elicited by the vaccine, as the level of specific antibodies correlates with protection against clinical disease. This threshold is critical to ensuring the vaccine elicits a sufficiently robust immune response to prevent viral replication and the onset of disease upon exposure to field strains. Although resource-intensive and requiring high-containment laboratory conditions, the PD₅₀ and PGP tests remain the gold standard for *in vivo* potency assessment and are vital for ensuring the consistency, efficacy, and field performance of FMD vaccines (30).

PGP and PD₅₀ tests, play a central role in evaluating and validating the protective capacity of FMD vaccines under standardized conditions. These tests serve as an important measure of the vaccine’s ability to confer protection under conditions that simulate real-world exposure to the virus (31). The standards established by the WOAH which mandate the use of the PGP and PD₅₀ tests, are essential for the regulatory approval of FMD vaccines, ensuring that vaccines meet stringent standards for both immunological effectiveness and clinical protection (32). The application of these standards plays a crucial role in the success of vaccination programs, contributing significantly to the control and potential eradication of FMD in livestock populations. Furthermore, regular monitoring of vaccine potency throughout its shelf life, using WOAH endorsed protocols is equally important to maintain efficacy and safeguard global livestock industries against FMD (33).

While *in vivo* challenge tests have long been regarded as the gold standard for assessing the potency of FMD vaccines, their application is not without significant limitations. Ethical concerns are a prominent drawback, as these tests often involve exposing animals to highly contagious and virulent pathogens, potentially resulting in severe illness or death (33). As ethical and welfare standards in animal research continue to tighten, the scale and scope of *in vivo* studies have become more restricted, necessitating the development of alternative, more humane testing approaches (25). In this context, the principles of the 3Rs have become central to the evolving framework for vaccine testing. As outlined by Grimm et al. (27), the principle of replacement advocates for the use of non-animal methods wherever feasible, such as *in vitro* models, computational simulations, and organ-on-a-chip technologies, which aim to mimic *in vivo* conditions without involving live animals. Reduction seeks to minimize the number of animals used in research, achieved through improved experimental designs, more efficient statistical methods, and the adoption of high-throughput technologies that enable the evaluation of multiple vaccine candidates with fewer animals. Refinement focuses on the optimization of experimental protocols to minimize animal suffering and enhance welfare outcomes. Strategies include improving vaccine challenge models to reduce disease duration and severity, adopting less invasive techniques, and implementing reliable early indicators of disease to facilitate timely intervention and the use of humane endpoints.

## 3 *In vitro* methods for assessing FMD vaccine immunogenicity and supporting potency evaluation

The ethical considerations surrounding *in vivo* testing are further compounded by the inherent species-specific variability in immune responses to FMDV. There is considerable variation in how different cloven-hoofed species respond immunologically to infection, and additional variability exists within the same species due to factors such as age, breed, and genetic predisposition (34, 35). This variability limits the ability of *in vivo* challenge tests to provide consistent and predictive data on vaccine efficacy across different species and populations, making them less reliable for assessing cross-species protection (36). Moreover, *in vivo* challenge testing is resource-intensive, requiring specialized facilities, skilled personnel, and stringent biosecurity measures to prevent the spread of FMDV within the research environment (37). These logistical and financial demands contribute to the high costs and extended time frames associated with such studies. In addition, the need for controlled challenge experiments and prolonged post-challenge monitoring of vaccinated animals further limit the utility of *in vivo* tests, particularly in situations requiring rapid screening of multiple vaccine candidates, such as during FMD outbreaks (38). These limitations underscore the necessity of developing alternative methods that can reduce animal use, improve efficiency, and address ethical concerns, all while maintaining the rigorous standards required to assess vaccine efficacy. Within the 3Rs framework, *in vitro* potency assays provide a replacement for *in vivo* testing by quantifying vaccine strength through measures such as viral binding, cytopathic effect inhibition, or viral load, thereby enabling ethical and precise evaluation of vaccine products. In contrast, *in vitro* immunogenicity and virus detection assays primarily provide readouts of immune responses rather than product potency. These assays can be applied in epidemiological studies, candidate vaccine evaluation, or as non-invasive endpoints in controlled vaccination studies, contributing to the refinement of *in vivo* testing by reducing reliance on severe clinical outcomes such as disease or death. Together, these approaches form a complementary landscape of tools for assessing FMD vaccine performance, balancing ethical considerations with scientific rigor and practical applicability. The continued refinement of vaccine testing protocols, in line with the 3Rs, is essential for advancing more ethical and sustainable approaches to FMD vaccine evaluation (27).

The limitations and ethical concerns associated with traditional *in vivo* methods for vaccine potency testing have led to a growing interest in developing alternative *in vitro* approaches for FMD vaccine evaluation (39, 40). *In vitro* techniques such as cell-based assays, molecular-based platforms, and ELISA are primarily designed to assess specific components of the immune response, such as neutralizing antibody activity, antigen-specific antibody production, and molecular markers of infection or immune activation (33). As such, these methods provide valuable insights into vaccine immunogenicity, rather than direct measures of product potency as defined in regulatory frameworks. Despite this distinction, *in vitro* approaches are increasingly recognized for their role in providing immunological readouts that can be used alongside established *in vivo* methods, particularly as non-invasive endpoints that support refinement of vaccine evaluation strategies. These methods offer the potential for improved biosafety, ethical acceptability, faster turnaround times, and the potential for high-throughput screening of vaccine performance. Nevertheless, the adoption of *in vitro* methods remains constrained by scientific validation requirements, regulatory acceptance, and practical implementation challenges, despite ongoing technological improvements (41).

A significant challenge in the adoption of *in vitro* methods is the absence of universally validated protocols. While regulatory frameworks such as the U.S. Code of Federal Regulations (CFR), Title 9, Part 113 provide standardized guidelines for certain potency and quality control tests, many *in vitro* methods remain in the early stages of validation and lack broad regulatory acceptance (42). Compared to *in vivo* methods, which have undergone extensive standardization over time, *in* vitro platforms often exhibit significant variability in experimental design, reagent composition, and instrumentation. These inconsistencies can substantially influence assay outcomes, complicating data interpretation, and limiting broader regulatory acceptance (43). As a result, it becomes difficult to ensure consistency across laboratories, hindering the ability to compare results and establish universally accepted benchmarks. While inter-governmental bodies such as the WOAH have recognized the potential of *in vitro* methods, they have not fully endorsed them as replacements for traditional *in vivo* potency tests for FMD vaccines. According to the WOAH Terrestrial Manual (21), *in vitro* methods may be used within vaccine evaluation frameworks provided they undergo rigorous validation and demonstrate analytical robustness, biological relevance, and reproducibility across laboratories. Importantly, while the manual does not exclude the use of *in vitro* assays, their acceptance remains conditional on the availability of robust supporting data. This includes a validated correlation between assay results and protective immunity, as determined in the target species.

*In vitro* methods have gained increasing regulatory attention and scientific support; however, their role is best understood as complementary rather than substitutive particularly for FMD vaccines. The complexity of the immune response to FMD virus and the variability in vaccine formulations necessitate disease-specific validation to ensure that any proposed *in vitro* method reliably reflects the vaccine’s protective efficacy. Although *in vivo* and *in vitro* assessments both demand significant resources, *in vitro* assays offer substantial advantages, including improved biosafety, ethical acceptability, and the potential for high-throughput screening of vaccine candidates or formulations (44). The subsequent sections shall provide a detailed overview of the main *in vitro* methodologies currently employed in FMD vaccine evaluation. Each method is examined in relation to its immunological basis, technical features, limitations, and recent innovations aimed at enhancing accuracy, scalability, and regulatory compliance.

### 3.1 Neutralization assay (virus neutralization test-VNT)

**Figure 2:**
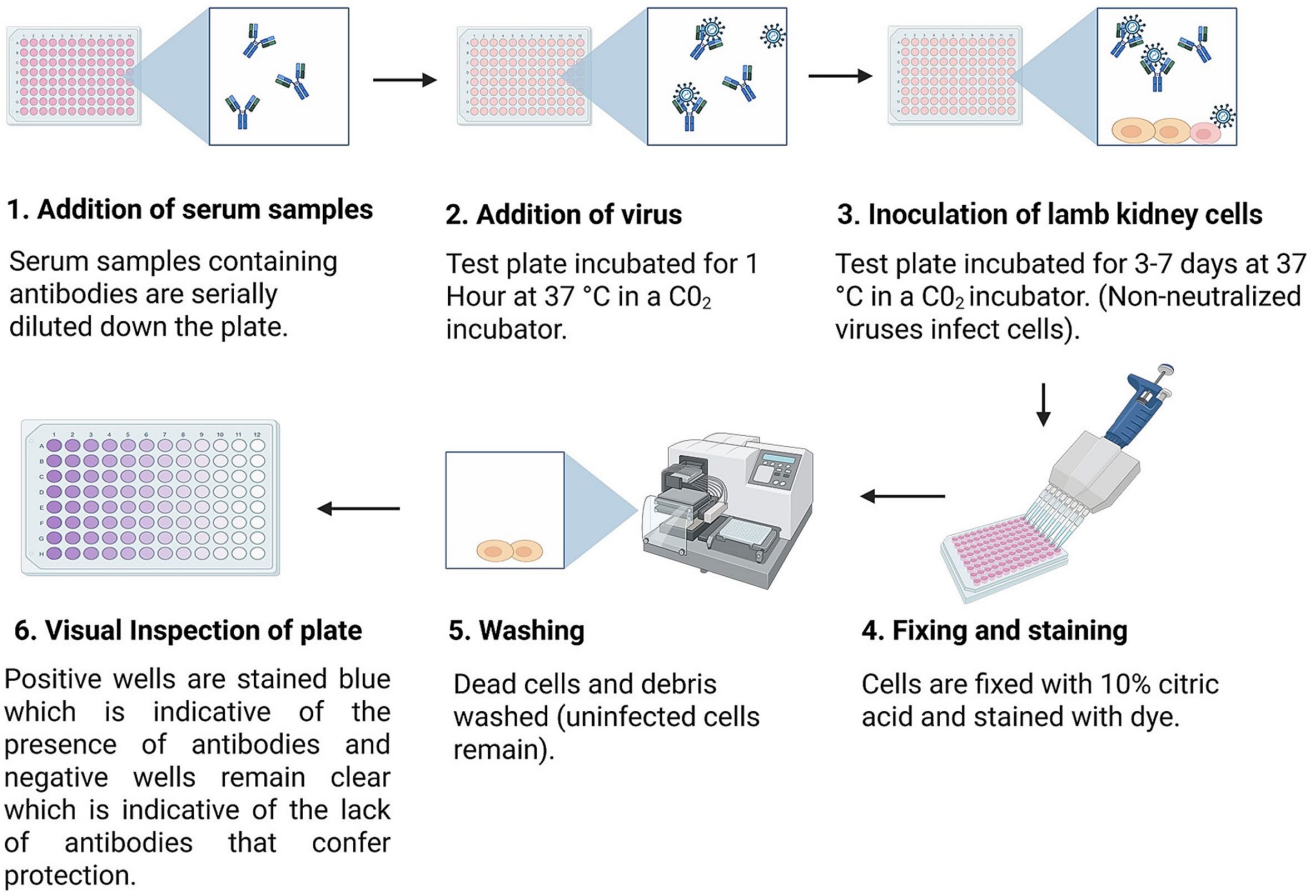
Schematic of the virus neutralization test (VNT) assay workflow. This diagram illustrates the sequential process of detecting functional, virus-neutralizing antibodies in test samples. In this procedure, serum samples from previously vaccinated or FMDV-exposed animals are serially diluted or titrated down the multi-well culture plate to assess the level of neutralizing antibodies. A standardized reference virus antigen, typically derived from field isolates propagated in permissive cell lines such as BHK-21 (baby hamster kidney) or lamb kidney cells is added to each well at a defined infectivity dose, commonly 100 TCID_50_ per well. The degree to which the serum antibodies neutralize the virus reflects the immune response elicited by vaccination or infection. In the context of vaccine evaluation frameworks, VNT remains a gold standard, as it quantitatively measures the functional antibody response and helps determine whether a vaccine induces a sufficient level of protective immunity.

The virus neutralization test (VNT) is one of the most commonly employed assays for assessing vaccine-induced humoral immunity in the context of FMD (45). It measures the ability of serum antibodies to inhibit viral infection in cell culture, thereby providing a functional *in vitro* assessment of neutralizing antibody activity. In this assay (Figure 2), serum samples are collected from animals that have been experimentally vaccinated under controlled conditions. These serum samples are then incubated with a standardized, titrated dose of infectious FMDV strain. The virus-serum mixture is subsequently applied to an infection-permissive cell culture system to evaluate residual viral infectivity. The neutralization titer, defined as the highest serum dilution that inhibits detectable cytopathic effects (CPE), provides a quantitative measure of the neutralizing antibody activity (46).

The VNT has been extensively validated through its application in diverse vaccine evaluation studies. For example, in a controlled potency study of a heptavalent oil-adjuvanted FMD vaccine in Egyptian calves, VNT titers measured at 28 days post-vaccination indicated measurable neutralizing antibody responses across multiple serotypes, with reported associations between titer levels and protection following live virus challenge (47). Similarly, a study evaluating a high-potency multivalent vaccine against A/ASIA/G-VII lineage in cattle reported associations between neutralizing antibody titer and clinical outcomes following viral challenge, with statistical analyses indicating a dose-dependent relationship between titer levels and the probability of protection (48).

VNT is widely recognized for its high specificity and its ability to provide a measure of antibody activity. However, while neutralizing antibody titers are often associated with protection, they do not constitute a direct measure of product potency or protective efficacy. Rather, they serve as an important immunological correlate that should be interpreted alongside other functional *in vivo* data (49). VNT, however, offers an ethical advantage by reducing the need for live-animal challenge studies, as it provides robust immunological data through *in vitro* testing. However, as noted by Gray et al. (50) despite its strengths, the assay presents several limitations: it requires specialized cell lines and carefully controlled laboratory conditions, making it labor-intensive and time-consuming. Moreover, VNT exclusively measures antibody-mediated neutralization, neglecting other critical components of the immune response, such as cell-mediated immunity, which also contributes significantly to protective efficacy (51).

Cell-mediated immunity, primarily involving T lymphocytes, plays a pivotal role in the control and clearance of viral infections (52). CD8^+^ cytotoxic T cells directly recognize and eliminate virus-infected cells, thereby limiting viral replication, while CD4^+^ helper T cells coordinate and enhance immune responses by supporting B cell function and sustaining cytotoxic T cell activity (53). These cellular responses are particularly important when neutralizing antibodies are insufficient, short-lived, or when viral variants partially evade antibody recognition.

Because VNT does not capture these cellular responses, additional assays such as interferon-*γ* ELISpot, intracellular cytokine staining, and T-cell proliferation assays are often used to provide complementary information on cellular immunity (54, 55). The integration of humoral and cellular immune assays enables a more comprehensive evaluation of vaccine-induced immune responses. Although VNT remains a widely used reference method for assessing neutralizing antibodies, its application is limited by biosafety requirements and relatively low throughput, which constrain its scalability. To address these challenges, advances such as assay automation and high-throughput adaptations have been developed to improve sample processing capacity an support more scalable application, while also improving consistency across experiments (56).

One particularly impactful innovation is the use of pseudotyped virus systems, in which replication-deficient viral vectors such as vesicular stomatitis virus (VSV) or lentiviruses are engineered to express FMDV surface proteins (57, 58). These systems preserve the antigenic properties necessary for immune recognition while eliminating the biohazard risks associated with handling live viruses. Conducted under standard biosafety level 2 (BSL-2) conditions, pseudovirus-based assays offer advantages in safety and scalability, as they can be implemented in a wider range of laboratory settings and are more amenable to high-throughput formats compared to assays requiring live virus (59). While pseudotyped systems provide practical benefits, they do not undergo full replication and therefore may not entirely mimic the complexity of natural infection. Ongoing advancements in pseudotyping technology have substantially improved their reproducibility, and overall performance. While their production requires specialized expertise and infrastructure, these systems are increasingly being recognized for their potential to complement traditional assays, particularly in the context of modern vaccine evaluation where rapid, high-throughput, and safe methodologies are in growing demand (60).

### 3.2 Enzyme-linked immunosorbent assay

**Figure 3:**
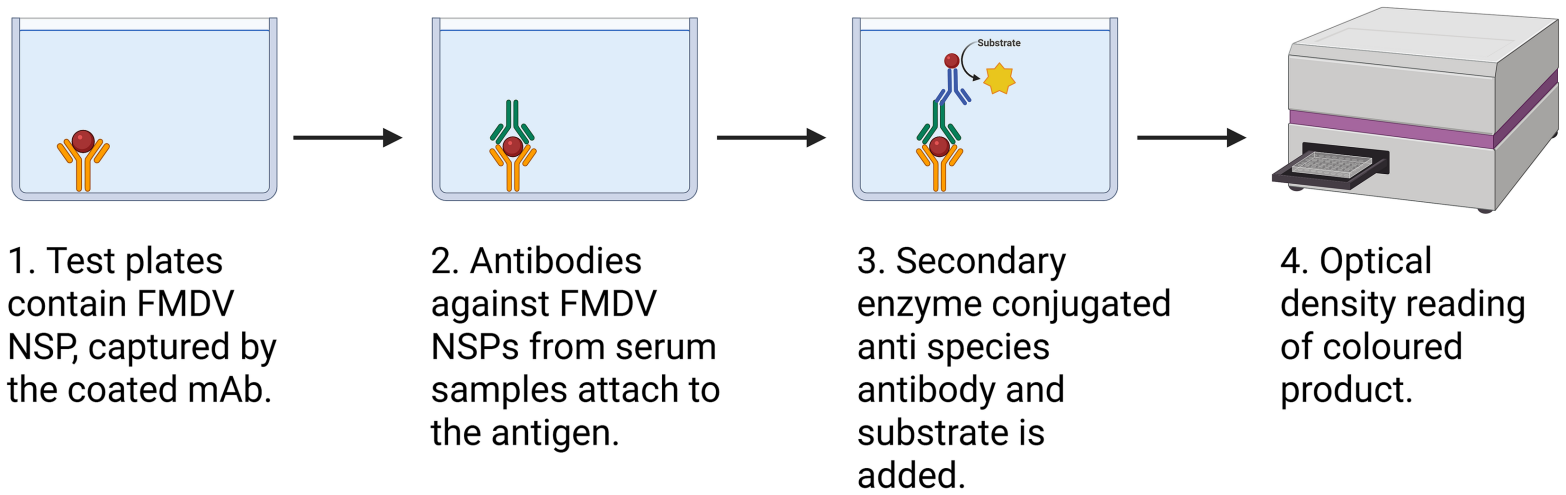
Stepwise illustration of the DIVA ELISA used to detect antibodies against FMDV non-structural proteins (NSP). The illustration depicts interactions in a multi-well culture plate pre-coated with FMDV NSP antigen, captured by a 3ABC-specific monoclonal antibody. Serum samples from test animals are added and may contain antibodies against the 3ABC antigen if the animal has been naturally infected with FMDV. These antibodies compete with a HRP-labeled detection monoclonal antibody for binding to the immobilized antigen. Following washing steps, the chromogenic substrate 3,3′,5,5′-tetramethylbenzidine (TMB) is added. The resulting color intensity measured by absorbance at 450 nm reflects the concentration of anti-NSP antibodies in the serum (91). Within vaccine evaluation and surveillance frameworks, NSP antibody ELISAs are crucial for distinguishing between vaccinated and infected animals, particularly in regions using purified, inactivated vaccines that do not elicit NSP responses. This differentiation is essential for accurately monitoring vaccine performance and verifying that serological responses result from vaccination rather than field virus exposure, thereby ensuring the reliability of potency assessments in FMD control programs.

Enzyme-linked immunosorbent assay (ELISA) is an immunological technique designed to detect and quantify specific antibodies or antigens in biological samples. In the context of FMD vaccine evaluation, ELISA is primarily applied to assess vaccine-induced humoral immune response, rather than direct product potency, by measuring antigen-specific antibodies induced by immunization (61). The assay, as shown in Figure 3, typically involves FMDV purified viral 3ABC antigens, which are captured by anti-3ABC monoclonal antibodies pre-coated onto the surface of a microtiter plate. Incubation of the antigen–antibody complex with serum from vaccinated animals allows antigen-specific antibodies to bind to the immobilized antigen, thereby distinguishing between infected and vaccinated individuals (DIVA). Following incubation, unbound components are removed through a series of washing steps, and a secondary antibody conjugated to an enzyme such as horseradish peroxidase (HRP) or alkaline phosphatase (AP) is introduced. This secondary antibody binds to the Fc-region of the antigen-specific antibodies. Upon the addition of a suitable chromogenic or fluorogenic substrate such as tetramethylbenzidine (TMB), an enzymatic reaction generates a measurable colorimetric or fluorescent signal, which is directly proportional to the concentration of bound antibodies in the sample (62).

ELISA has been widely employed in both controlled and field-based studies to monitor FMD vaccine-induced immune response. For example, in a field trial conducted among cattle herds in Zambia, the assay was used to quantify post-vaccination antibody responses in animals administered with either a single or double dose of a commercial trivalent FMD vaccine (63). The findings demonstrated that animals receiving booster vaccination exhibit significantly higher antibody titers and greater rates of seroconversion compared to single-dose regimens. Similarly, in a sero-monitoring study of Ethiopian livestock, ELISA was employed to track antibody kinetics over 120 days following immunization. Antibody levels were maintained within this timeframe, particularly in animals vaccinated with an oil-adjuvanted polyvalent vaccine; however, titers declined by later time points, reflecting the relatively short-lived or limited immunity associated with inactivated FMD vaccines, underscoring the need for regular booster vaccination in endemic areas (64).

ELISA offers several advantages that make it a widely adopted platform for immunological assessments in both research and clinical settings. The advantages include; high analytical sensitivity when high affinity antibodies are used, rapid execution, and the capacity to process in a high-throughput format (65). When appropriately standardized, ELISA can provide semi-quantitative or quantitative data on antigen-specific antibody responses, which are often used to assess vaccine immunogenicity and monitor sero-conversion following vaccination (60). However, the assay has inherent limitations that must be considered when interpreting results. Most notably, ELISA is restricted to the detection of humoral immune responses and does not capture the contributions of cell-mediated immunity, which is an essential component of protective immunity against many viral pathogens. As such, reliance solely on ELISA data may lead to an incomplete assessment of vaccine efficacy. Furthermore, ELISA is susceptible to cross-reactivity, particularly when closely related viral antigens or conserved epitopes are present across different pathogens or vaccine strains. Pre-existing antibodies generated from prior infections or vaccinations may bind to shared or conserved epitopes on the coated antigens, resulting in cross-reactive signals. This can lead to overestimation of antigen-specific antibody responses and complicate the interpretation of serological data, particularly in endemic settings or in animal populations with diverse exposure histories (15). Therefore, while ELISA remains a valuable tool for serological evaluation, its results should ideally be complemented with functional assays and cellular immune profiling for a more comprehensive understanding of vaccine-induced immune responses.

Advances in ELISA technology have improved assay performance through the use of monoclonal antibodies, which provide high specificity and reproducibility when used as capture reagents (60). Meanwhile, secondary antibodies are typically used as polyclonal pools to enable recognition of multiple epitopes, thereby amplifying the detection signal and enhancing assay sensitivity. In sandwich ELISA formats, this may involve a detection antibody binding to the captured antigen, followed by an enzyme-conjugated anti species antibody that further amplifies the signal. Although monoclonal secondary antibodies are available, they are less frequently used due to typically lower signal amplification. Together, these improvements in antibody design and selection have resulted in more versatile and adaptable ELISA detection methods (62). In parallel, Tabatabaei et al. (61) noted that integration of nanotechnology into immunoassay design has further augmented both the sensitivity and specificity of ELISAs. Nanoparticle-enhanced assays, which employ materials such as gold nanoparticles or quantum dots, serve to amplify signal detection and facilitate the identification of low-abundance antibodies. These nanoparticles can be functionalized to selectively bind to specific immunoglobulin isotypes or antigen–antibody complexes, enabling detection at significantly lower concentrations than conventional methods. Such innovations not only improve analytical sensitivity but also contribute to faster, more cost-effective, and robust immunoassays. Collectively, these technological improvements position ELISA and related platforms as increasingly offering the potential to improve vaccine evaluation, reduce reliance on animal testing, and enhance the overall efficiency of disease control efforts (66).

Recombinant antigen-based ELISAs, which rely on laboratory-produced viral proteins rather than whole or live viruses represent another important advancement, offering improved biosafety, reduced reliance on live virus materials, and greater adaptability across virus types or serotypes (67). However, despite these benefits, recombinant antigen-based ELISAs have notable limitations. Since recombinant proteins may not perfectly mimic the conformational structure of native viral antigens, the assays can suffer from reduced specificity and sensitivity. Moreover, like most serological tests, they are limited to measuring humoral (antibody-mediated) immune responses and do not capture cell-mediated immune responses, which play a crucial role in protective immunity (68). To overcome these limitations, molecular techniques such as reverse transcription quantitative polymerase chain reaction (RT-qPCR) are increasingly being integrated into immunoassay workflows to provide complementary insights into infection status and immune responses. While ELISA enables the detection and quantification of vaccine-induced antibody responses, RT-qPCR facilitates the sensitive detection of viral RNA, particularly during early stages of infection. Studies have demonstrated that the combined use of ELISA and RT-qPCR enhances the accuracy and reliability of FMD diagnosis and monitoring, thereby improving the overall evaluation of vaccine performance (69).

### 3.3 Cytotoxicity or cytopathogenicity assays

**Figure 4:**
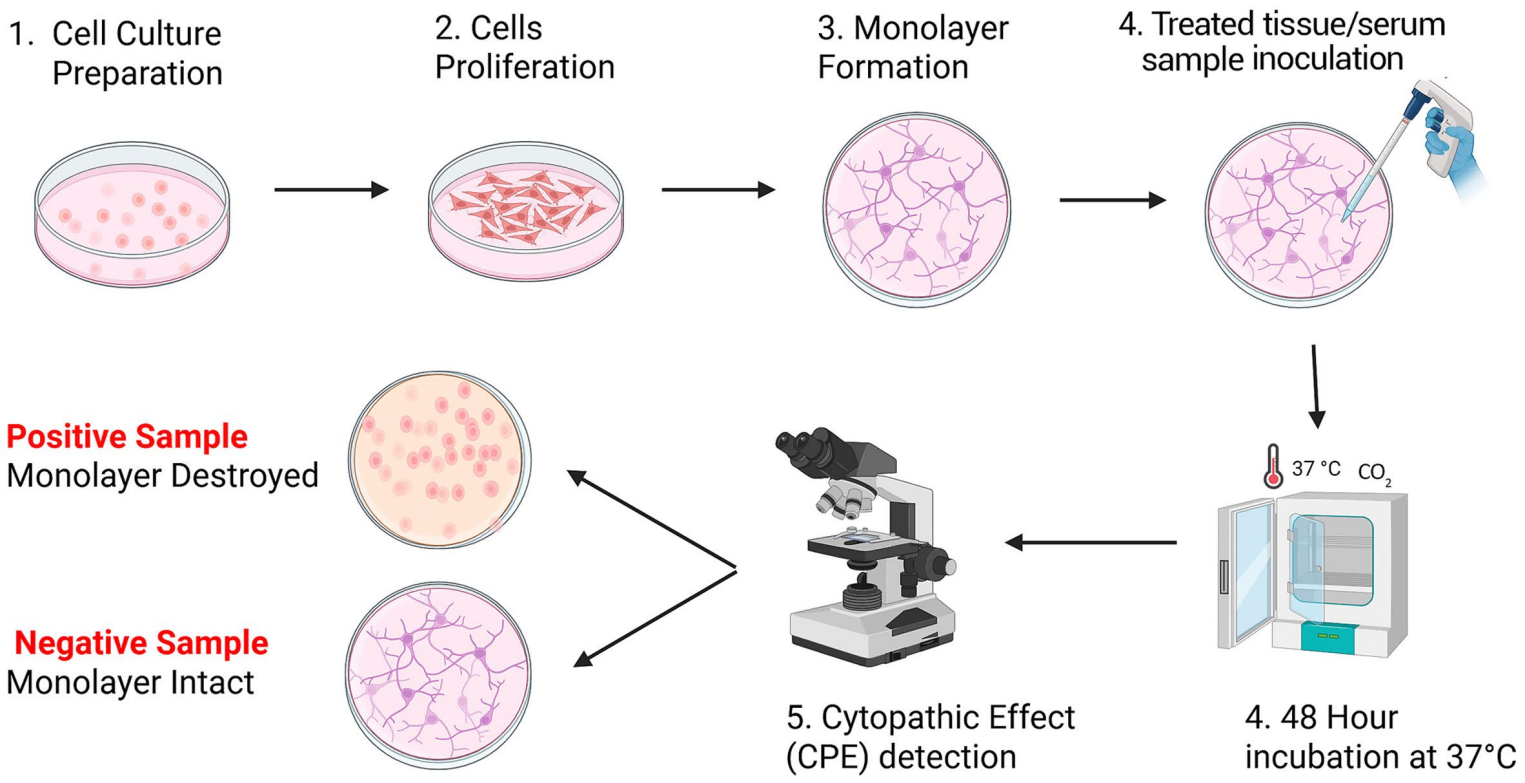
Schematic representation of cell-based (cytotoxicity or cytopathogenicity) assays. This illustration depicts the process of detecting FMDV in tissue and serum samples using infection-susceptible lamb kidney primary cells cultured in growth media. The procedure entails cell culture preparation and proliferation in flasks to a confluent monolayer. Epithelial tissue, probang (oropharyngeal fluid) or serum samples which may contain FMD virus are inoculated onto the cell monolayer and incubated. The plates are examined under a microscope after 48 h to determine the presence of CPE. The presence of the virus would destroy the monolayer due to viral replication whereas the absence of the virus would be identified by an intact monolayer.

Cytotoxicity or cytopathogenicity assays are cell-based methods widely used to evaluate the functional activity of vaccine-induced antibodies, particularly their ability to neutralize viral infection *in vitro* (70). In the context of FMD vaccine evaluation, these assays primarily assess neutralizing antibody responses, rather than direct product potency, by measuring the capacity of serum antibodies to inhibit virus replication in susceptible cell cultures. These assays, as illustrated in Figure 4, typically involve co-incubating serum samples from vaccinated animals with FMDV, followed by infection of FMDV-susceptible cell lines such as Vero or BHK-21 cells. Neutralizing antibodies present in the serum bind to the virus and inhibit its ability to infect cells. The extent to which serum-derived antibodies neutralize the virus as evidenced by reduced viral replication or attenuation of CPE serves as a direct measure of vaccine-induced neutralizing antibody activity, a principle similarly demonstrated in cytopathogenicity-based neutralization assays for SARS-related coronaviruses (71).

Cytotoxicity and cytopathogenicity assays provide a biologically relevant measure of antibody functionality by linking immune responses to the inhibition of viral replication at the cellular level. Combining morphological assessment of CPE with measurement of cell viability enables detailed analysis of virus-host interactions and antibody-mediated neutralization (72). The resulting data offer critical insight into viral pathogenesis and provide a functional measure of the immune responses elicited by vaccination. In addition to evaluating immune protection, cytotoxicity and cytopathogenicity assays are frequently used as platforms for antiviral drug screening and for the pre-clinical evaluation of vaccine candidates under *in vitro* conditions that closely resemble physiological infection. The methodological versatility of these assays underpins their broad application in vaccine research, viral surveillance and functional evaluation of immune responses (25).

Cell-based neutralization assays are essential tools for evaluating the functional antibody response elicited by FMD vaccines. In a potency assessment of a heptavalent oil-adjuvanted FMD vaccine conducted in Egypt, BHK-21 cell-based neutralization assays were employed to quantify the ability of post-vaccination sera to inhibit virus-induced cytopathic effects (CPE) over a 48-h period (47). Sera from vaccinated animals effectively prevented visible CPE at specific dilutions, thereby confirming the presence of neutralizing antibodies. Importantly, such assays provide a functional correlate of immune response without requiring live-animal challenge studies, thereby supporting Refinement of *in vivo* testing approaches. In addition, Browne et al. (73) further noted that supplementing cell-based assays with RT-qPCR significantly enhanced the sensitivity and precision of immune response measurements. RT-qPCR enables sensitive and quantitative detection of viral RNA, including at very low levels, thereby providing a complementary measure of visual CPE-based assessment of viral activity (74). This combined approach improves the resolution of neutralization measurements and supports more accurate evaluation of vaccine-induced immune responses. As a result, researchers and health authorities can initiate timely interventions, such as revaccination or vaccine reformulation, thereby improving the overall effectiveness of disease control strategies. However, despite these advantages, cell-based assays are not without limitations. The results can be influenced by the choice of cell lines, as different cell types may exhibit varying susceptibility to viral infection and differential responses to neutralizing antibodies. Factors such as receptor expression, cellular metabolism, and cell-intrinsic antiviral response pathways can all affect assay outcomes, potentially leading to variability in the interpretation of vaccine efficacy (75).

Traditional two-dimensional (2D) cell culture systems, while useful, often fail to accurately replicate the complex biological interactions that occur in living organisms, posing challenges for reliably assessing vaccine efficacy *in vitro*. To address these challenges, significant advancements have been made. The development of three-dimensional (3D) cell culture models and organoid systems has improved the biological relevance of *in vitro* platforms, offering more accurate simulations of host–pathogen interactions (76). These systems better mimic tissue architecture, cellular heterogeneity and microenvironmental conditions, enabling more representative modeling of viral infection dynamics and immunity. Fang and Eglen (77) further supported this by demonstrating that, in the context of viral infections such as FMD, 3D cultures can simulate the spatial and temporal dynamics of virus–host interactions, immune responses, and tissue damage more realistically than flat monolayers. Organoid systems are miniaturized, self-organizing structures derived from stem cells that mimic the architecture and function of specific organs. These models are particularly useful for studying tissue-specific responses to infection and for evaluating the efficacy and safety of vaccines in a controlled, yet complex, *in vitro* environment (78).

In parallel, the integration of automated technologies and high-throughput screening platforms has significantly increased the efficiency and scalability of cell-based assays (78). Automation reduces human error, standardizes procedures, and enables precise control over assay conditions, leading to improved reproducibility across experiments and laboratories (79). High-throughput systems allow for the simultaneous evaluation of multiple samples, which is particularly advantageous during early-phase vaccine development and large-scale screening campaigns. This capability accelerates the identification of promising vaccine candidates by enabling rapid assessment of immune responses, cytopathogenic effects, and viral neutralization across different formulations and dosages (48). Zhu (78) also affirmed that together, these innovations not only improve the physiological relevance and throughput of *in vitro* testing but also contribute to reducing reliance on animal models, supporting more ethical and streamlined approaches in modern vaccine development.

### 3.4 Reverse genetics and molecular-based assays

**Figure 5:**
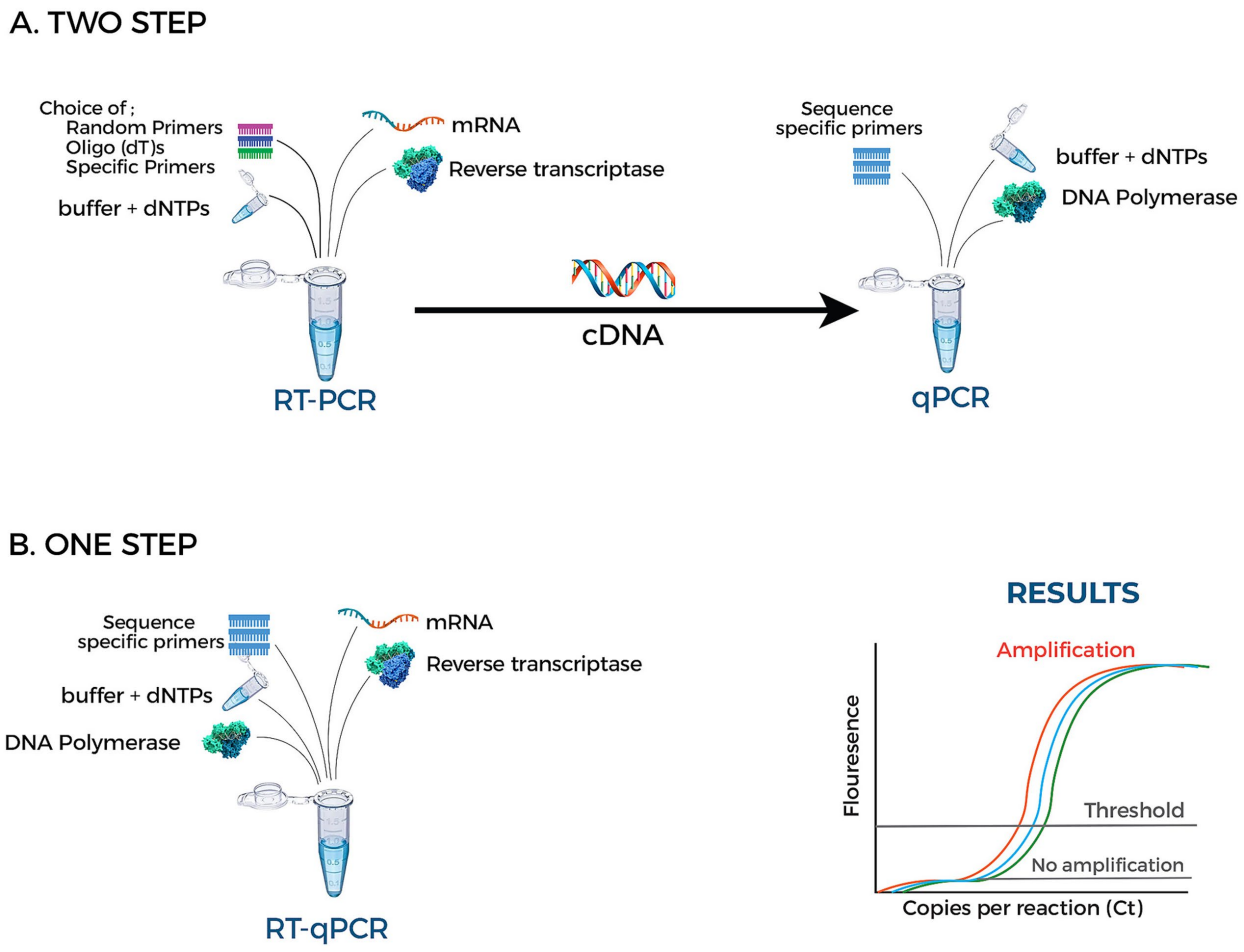
Schematic illustration of reverse transcription polymerase chain reaction (RT-PCR) workflows for FMD diagnosis. **(A)** Two-step RT-qPCR: Viral RNA is first reverse transcribed into complementary DNA (cDNA) using reverse transcriptase and appropriate primers, followed by amplification in a separate quantitative PCR (qPCR) reaction using sequence-specific primers and DNA polymerase. **(B)** One-step RT-qPCR: Reverse transcription and qPCR amplification are performed simultaneously in a single reaction tube containing reverse transcriptase, DNA polymerase, primers, and reaction buffer components. The amplification curves illustrate fluorescence-based detection during qPCR, with cycle threshold (Ct) values inversely related to viral RNA concentration.

Molecular-based techniques, particularly reverse genetics and nucleic acid amplification methods such as conventional polymerase chain reaction (PCR), reverse transcription PCR (RT-PCR), and reverse transcription quantitative PCR (RT-qPCR), are widely used for detecting and quantifying viral nucleic acids and assessing host transcriptional responses in FMD research. In the context of vaccine evaluation, these methods are primarily applied to monitor viral RNA levels and host molecular responses, rather than directly assess functional immunity or product potency. However, nucleic acid amplification can also serve as an *in vitro* alternative to traditional *in vivo* potency assays. According to Ranheim et al. (80), PCR-based approaches can enable sensitive and quantitative detection of genomes at defined time points in controlled assays, providing a molecular readout of vaccine performance in a dose–response framework. By detecting even minimal quantities of viral RNA, these methods offer precise and reproducible surrogate measures of vaccine potency within standardized *in vitro* assays. In FMDV studies, RT-qPCR-based approaches have been used to evaluate viral suppression in cell cultures exposed to vaccine-treated virus, allowing quantitative assessment of vaccine strength without the need for live-animal challenge (Figure 5). More broadly, PCR-based assays remain valuable for detecting low levels of viral RNA with high analytical sensitivity, supporting applications such as infection detection and virological monitoring in experimental and field settings. This high analytical sensitivity is especially important for assessing viral load during the initial stages of infection, when viral concentrations may be low but clinically relevant (81).

RT-qPCR is a widely used molecular diagnostic tool in both laboratory and field settings due to its sensitivity, specificity and reproducibility. For example Dong et al. (82) reported the development of a TaqMan RT-qPCR assay with a detection threshold of 64.3 copies/μL, demonstrating high analytical sensitivity for FMDV detection in clinical samples even in the presence of potential inhibitors. Beyond viral RNA quantification, molecular techniques such as digital droplet PCR, RNA sequencing, and gene expression profiling are increasingly used to assess host immune responses at the transcriptional level. These approaches allow for the measurement of immune-related gene expression, including cytokines, chemokines, and interferons, providing insights into the molecular mechanisms underlying vaccine-induced immune responses (81).

While molecular assays offer high sensitivity and quantitative capability, they do not directly assess functional immune responses, a limitation that becomes particularly important when interpreting vaccine-induced protection, as molecular assays detect viral RNA or gene expression but neither distinguish between infectious and non-infectious virus, nor do they capture the ability of the immune system to neutralize or eliminate the virus (81). In the case of inactivated FMD vaccines, PCR, RT-PCR, and RT-qPCR assays are primarily used to measure viral RNA load or detect immune-related transcripts, such as cytokines, as indirect indicators of vaccine-induced responses. In this context, they may provide useful early-stage insights into infection dynamics and immune activation (83). However, with the emergence of next-generation vaccines (NGVs) such as epitope-based, DNA, or mRNA vaccines, which are specifically designed to elicit strong cell-mediated immune responses, the ability of molecular assays to fully capture vaccine-induced immunity becomes more limited. Advanced vaccine technologies such as DNA, mRNA, and epitope-based vaccines aim to stimulate cytotoxic T-cell responses, enhance cytokine signaling, and establish long-term immunological memory (84). While RT-qPCR can measure the transcription of cytokine genes or immune markers, it does not capture functional cellular activity, such as the ability of T-cells to recognize and eliminate infected cells. To address this gap, recent innovations like single-cell RNA sequencing and bulk transcriptomics have allowed for more comprehensive profiling of immune responses at high resolution, providing insights into the heterogeneity and dynamics of vaccine-induced immunity (85). However, although these approaches offer improved resolution of gene expression profiles, they remain limited in their ability to directly assess functional immune activity, as they measure transcriptional outputs rather than cellular effector functions (81). This highlights the importance of integrating molecular assays with complementary techniques to obtain a more comprehensive and physiologically relevant evaluation of vaccine efficacy.

In addition to their interpretive limitations, molecular assays require access to specialized instrumentation such as real-time thermocyclers and trained personnel to ensure accuracy and consistency. Technical factors, including variations in RNA extraction, primer design, and reaction efficiency, can contribute to discrepancies between laboratories or experimental conditions, potentially affecting reproducibility (86). As a result, while molecular assays remain invaluable for their sensitivity, specificity, and speed, they are best used in combination with other immunological *in vitro* methods to achieve a more complete and functionally relevant assessment of vaccine-induced immune responses.

Recent advances in artificial intelligence (AI) and machine learning are increasingly being explored for improving diagnostics and vaccine evaluation in FMD. AI-based approaches have been applied to automate disease detection and monitoring. In particular, image-based systems have shown potential to distinguish between infected and protected animals, with high reported sensitivity and specificity (87). However, these models are typically developed under controlled conditions, and their performance may vary in field settings where environmental and biological variability can influence outcomes. Similarly, machine learning models have been reported to identify FMD outbreaks and high-risk regions (88), although their predictive performance is often dependent on region-specific datasets, which may limit broader applicability. Computational tools have also been developed to support the prediction of FMDV serotypes from sequence data (89), offering a potentially faster alternative to conventional methods, however, their accuracy is influenced by the quality and diversity of available sequence data. Overall, while these approaches highlight the potential of AI to enhance diagnostic and epidemiological capabilities, the current evidence remains context-dependent and requires further validation across diverse field settings.

## 4 Conclusion and future perspectives

The growing recognition of the ethical, economic, and scientific limitations of traditional *in vivo* testing has driven a significant shift toward *in vitro* approaches for evaluating FMD vaccine-induced immune responses. Although challenges concerning serotype diversity, immunological complexity, and regulatory acceptance remain, advancements in molecular biology, cell culture systems, and nanotechnology are enabling the development of more reliable, standardized, and ethically responsible *in vitro* assays. These innovations promise to transform FMD vaccine evaluation by offering faster, safer, and more reproducible alternatives to live-animal testing. Looking ahead, future efforts should prioritize the development of functionally relevant, serotype-transcending *in vitro* assays that reflect the breadth and depth of protective immunity. This is particularly important for FMDV, a virus known for its high diversity and antigenic variability. Equally important is the harmonization of assay protocols and validation standards across laboratories and regions, which would improve comparability of results and support global vaccine trade and deployment. Additionally, to ensure broad regulatory acceptance, there is a need for collaborative frameworks between researchers, vaccine manufacturers, and regulatory bodies aimed at aligning emerging technologies with established licensing criteria.

Ultimately, the future of FMD vaccine testing should be guided by the dual goals of scientific rigor and practical applicability. *In vitro* approaches must not only reduce ethical burdens and biosafety risks but also generate immune correlates that meaningfully predict real-world protection. By addressing both the technical and translational gaps, next-generation *in vitro* methods can play an important complementary role in controlling FMD outbreaks and strengthening global animal health systems.

## Glossary

AI: Artificial intelligence
AP: Alkaline phosphatase
BEI: Binary ethylenimine
BHK-21: Baby hamster kidney cell line
BSL-2: Biosafety level 2
CPE: Cytopathic effect
CQA: Critical quality attribute
CTL: Cytotoxic T lymphocyte
DIVA: Differentiating infected from vaccinated animals
DNA: Deoxyribonucleic acid
ELISA: Enzyme-linked immunosorbent assay
EU: European Union
FMD: Foot-and-mouth disease
FMDV: Foot-and-mouth disease virus
HRP: Horseradish peroxidase
LPBE: Liquid-phase blocking ELISA
MAC: Membrane attack complex
ML: Machine learning
mAb: Monoclonal antibody
NSP: Non-structural protein
pAb: Polyclonal antibody
PCR: Polymerase chain reaction
PD₅₀: 50% protective dose
PGP: Protective Group Proportion
RNA: Ribonucleic acid
RT-PCR: Reverse transcription polymerase chain reaction
RT-qPCR: Reverse transcription quantitative polymerase chain reaction
SAT: Southern African Territories
SP: Structural protein
TMB: Tetramethylbenzidine
VNT: Virus neutralization test
VSV: Vesicular stomatitis virus
WOAH: World Organisation for Animal Health
Vaccine potency: The ability of a vaccine to elicit measurable and protective response, typically assessed through immunogenicity or challenge studies
Vaccine efficacy: The degree to which a vaccine reduces disease incidence or severity under controlled experimental conditions, often determined through clinical or challenge trials
Vaccine safety: The absence of unacceptable adverse effects following vaccination, ensuring that the vaccine does not cause harm to the recipient
Critical quality attributes (CQAs): Measurable physical, chemical, biological or microbiological properties that must be controlled to ensure quality, safety, and efficacy of a vaccine
